# Supplementary material for: Development of a computer-aided design software for the quantitative evaluation of aesthetic damage
Source: PLoS One. 2019 Dec 18;14(12):e0226322. doi: 10.1371/journal.pone.0226322 (PMC6919621; doi:10.1371/journal.pone.0226322)
Supplement: S1 Code site — 1. (ZIP) [file pone.0226322.s001.zip › [EESC_jr][Projeto]Relatorio_07_21/reports/templates_zip/startbootstrap-grayscale-gh-pages.zip!/startbootstrap-grayscale-gh-pages/index.html]

Grayscale - Start Bootstrap Theme


Menu 

 Start Bootstrap

- About
- Download
- Contact


# Grayscale

A free, responsive, one page Bootstrap theme.
  
Created by Start Bootstrap.


## About Grayscale

Grayscale is a free Bootstrap 3 theme created by Start Bootstrap. It can be yours right now, simply download the template on the preview page. The theme is open source, and you can use it for any purpose, personal or commercial.

This theme features stock photos by Gratisography along with a custom Google Maps skin courtesy of Snazzy Maps.

Grayscale includes full HTML, CSS, and custom JavaScript files along with LESS files for easy customization.

## Download Grayscale

You can download Grayscale for free on the preview page at Start Bootstrap.

Visit Download Page

## Contact Start Bootstrap

Feel free to email us to provide some feedback on our templates, give us suggestions for new templates and themes, or to just say hello!

feedback@startbootstrap.com

- Twitter
- Github
- Google+


Copyright © Your Website 2016
